# Supplementary material for: Identification of Binding Proteins for TSC22D1 Family Proteins Using Mass Spectrometry
Source: Int J Mol Sci. 2021 Oct 9;22(20):10913. doi: 10.3390/ijms222010913 (PMC8536140; doi:10.3390/ijms222010913)
Supplement: Supplementary file 1 [file ijms-22-10913-s001.zip › Table S1.pdf]

Table S1. Mascot score and peptide match of the identified proteins from GST-TSC-22pull-down assay （TYS, whole cell extracts）

Protein Names	Mascot score	Peptide match
Heat shock 70 kDa protein 1A/1B	114	5
Keratin, type II cytoskeletal 1	256	20
Pleckstrin homology domain-containing family G member 2	15	2
Elongation factor 1-gamma	268	20
Rho-related GTP-binding protein Rho6	13	6
Carbonyl reductase [NADPH] 1	244	9

LC/MS/MS data were analyzed using Mascot software against SwissProt database. Identified proteins with the score higher than 13 were listed on the Table S1.
