# Supplementary material for: Identification of Binding Proteins for TSC22D1 Family Proteins Using Mass Spectrometry
Source: Int J Mol Sci. 2021 Oct 9;22(20):10913. doi: 10.3390/ijms222010913 (PMC8536140; doi:10.3390/ijms222010913)
Supplement: Supplementary file 1 [file ijms-22-10913-s001.zip › Table S2.pdf]

Table S2. Mascot score and peptide match of the identified proteins from *In vivo* Flag-TSC-22 binding assay（HEK293, nuclear extracts）

Protein Names	Mascot score	Peptide match
Histone H1.2	92	8
Lamin-B1	76	7
Guanine nucleotide-binding protein-like 3	36	4
Pituitary adenylate cyclase-activating polypeptide type I receptor	18	1
Heterogeneous nuclear ribonucleoprotein A1	53	3
Charged multivesicular body protein 4a	220	3
rRNA 2'-O-methyltransferase fibrillarin	41	1

LC/MS/MS data were analyzed using Mascot software against SwissProt database. Identified proteins with the score higher than 13 were listed on the Table S2.
