# Supplementary material for: Identification of Binding Proteins for TSC22D1 Family Proteins Using Mass Spectrometry
Source: Int J Mol Sci. 2021 Oct 9;22(20):10913. doi: 10.3390/ijms222010913 (PMC8536140; doi:10.3390/ijms222010913)
Supplement: Supplementary file 1 [file ijms-22-10913-s001.zip › Table S3.pdf]

Table S3. Mascot score and peptide match of the identified proteins from *In vivo* Flag-TSC22 (86) binding assay (HEK293, whole cell extracts)

Protein Names	Mascot score	Peptide match
Histone H1.2	62	6
40S ribosomal protein S6	129	4
Keratin, type II cytoskeletal 1	165	9
Keratin, type I cytoskeletal 9	147	7
Actin, cytoplasmic 1	318	27
Glyceraldehyde-3-phosphate dehydrogenase	36	2
Ubiquitin carboxyl-terminal hydrolase 47	27	2
Dermcidin	35	2
Protein tyrosine phosphatase receptor type C-associated protein	26	5
40S ribosomal protein S2	30	1

LC/MS/MS data were analyzed using Mascot software against SwissProt database. Identified proteins with the score higher than 13 were listed on the Table S3.
