# Supplementary figures and images for: Identification of Binding Proteins for TSC22D1 Family Proteins Using Mass Spectrometry
Source: Int J Mol Sci. 2021 Oct 9;22(20):10913. doi: 10.3390/ijms222010913 (PMC8536140; doi:10.3390/ijms222010913)

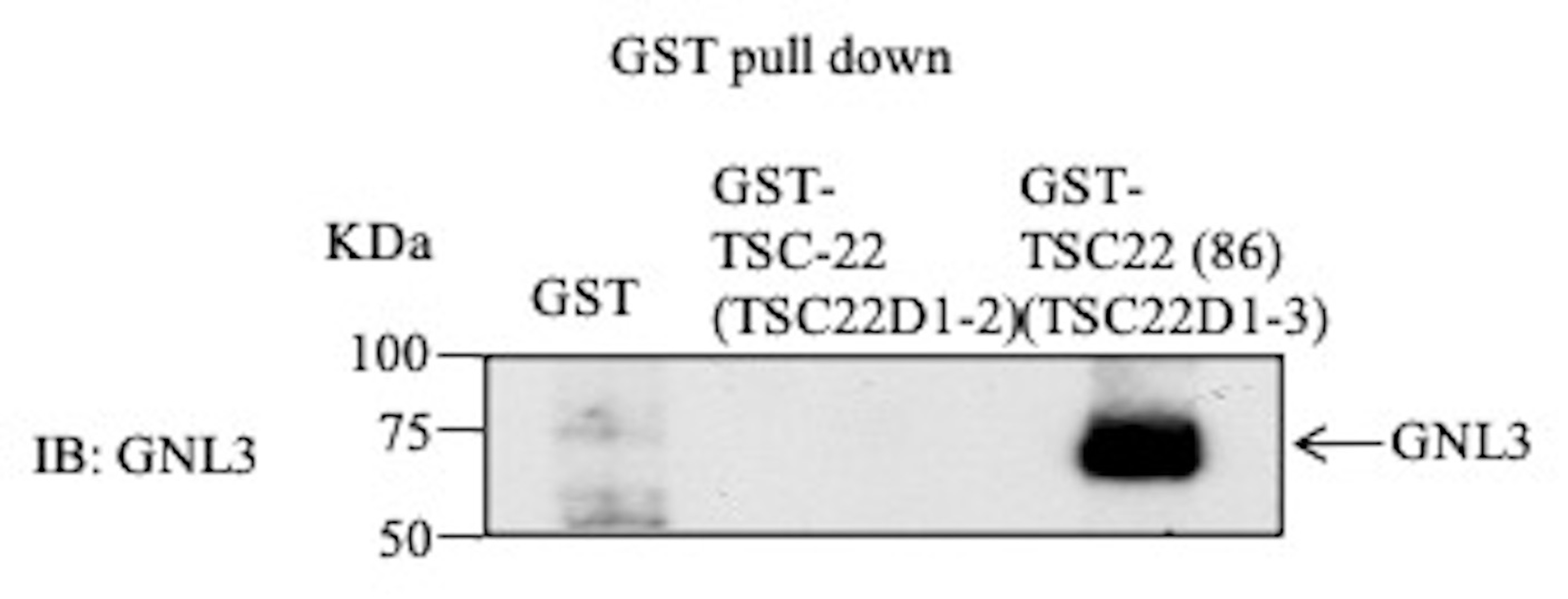

Supplement: Supplementary file 1 [file ijms-22-10913-s001.zip › FigS1.jpg]

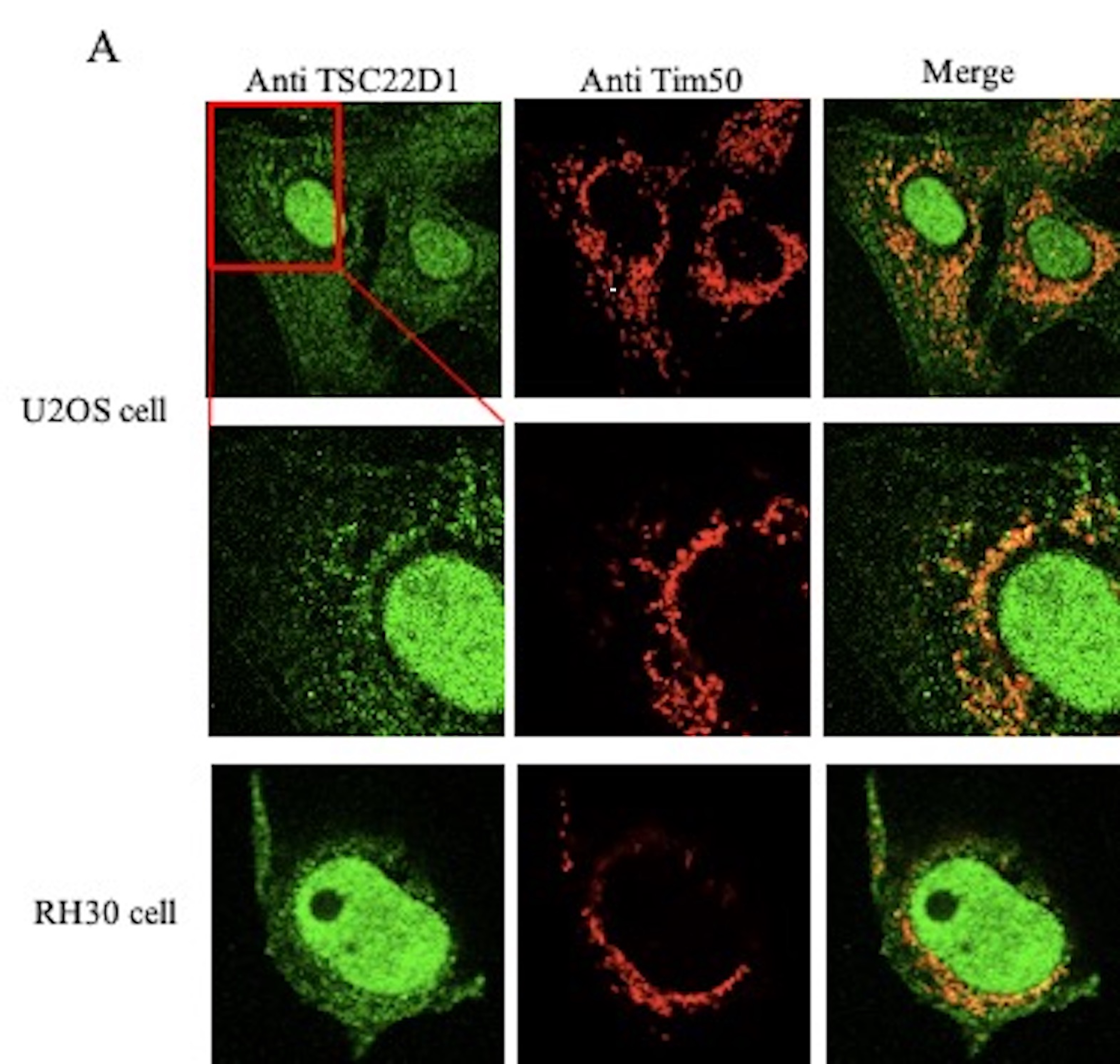

Supplement: Supplementary file 1 [file ijms-22-10913-s001.zip › FigS2A.jpg]

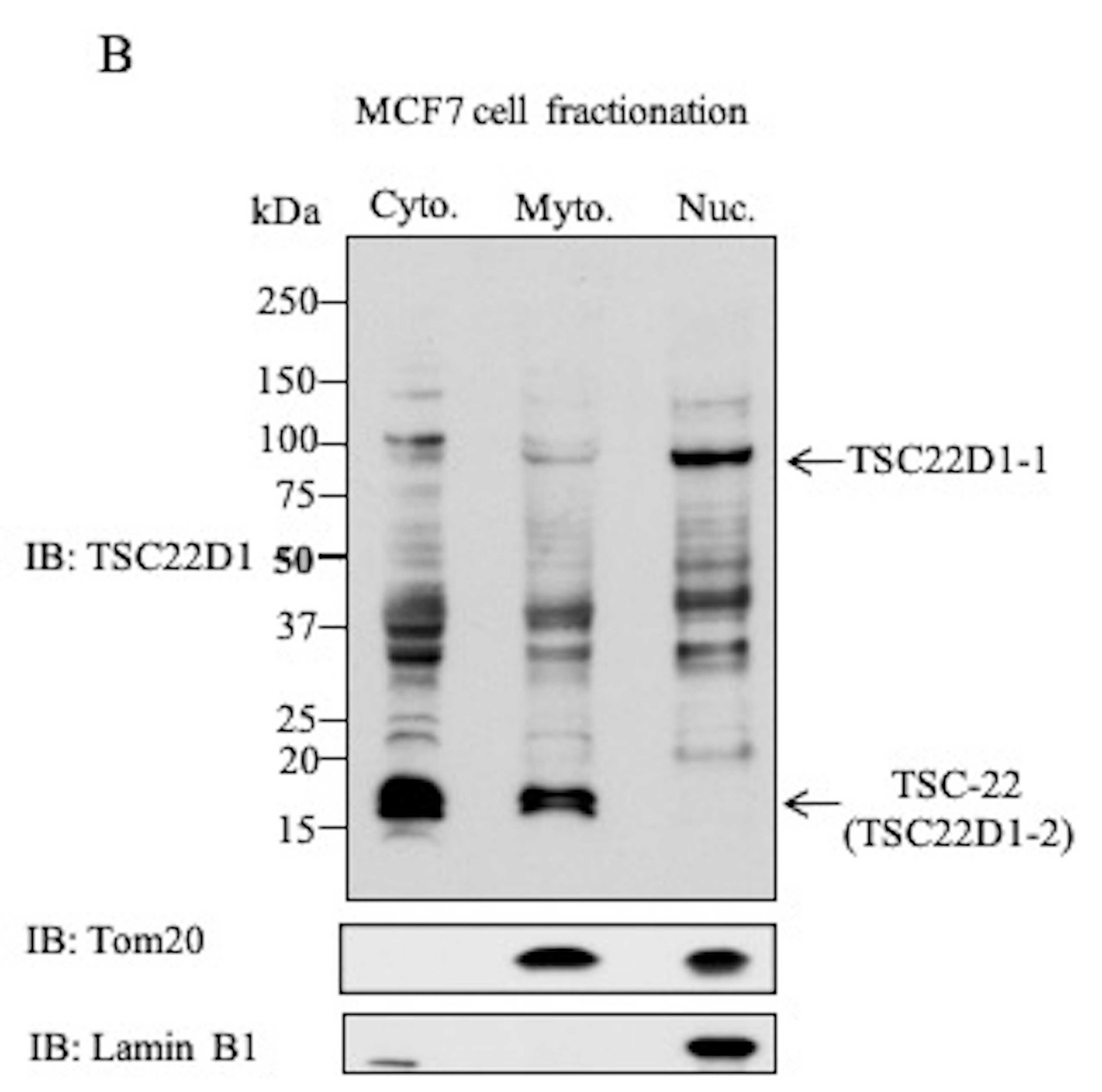

Supplement: Supplementary file 1 [file ijms-22-10913-s001.zip › FigS2B╕─.jpg]
